# *catena*-Poly[[[diaqua­manganese(II)]-μ_3_-pyridine-2,3-dicarboxyl­ato-κ^4^ *N*,*O* ^2^:*O* ^3^:*O* ^3′^] dihydrate]

**DOI:** 10.1107/S1600536808029413

**Published:** 2008-09-20

**Authors:** Zhong-Xiang Du, Jun-Xia Li

**Affiliations:** a Department of Chemistry and Chemical Engineering, Luoyang Normal University, Luoyang, Henan 471022, People’s Republic of China

## Abstract

In the title coordination polymer, {[Mn(C_7_H_3_NO_4_)(H_2_O)_2_]·2H_2_O}_*n*_, the Mn^II^ ion is coordinated in a distorted octahedral environment by the O atoms of two water mol­ecules, one N and one O atoms of the chelating pyridine-2,3-dicarboxyl­ate (PDC) dianion, and two axial bridging carboxyl­ate O atoms from two adjacent PDC ligands. The fully deprotonated PDC anion acts a μ_3_-bridging ligand, establishing a chain structure along the *a* axis. These polymeric chains are connected into a three-dimensional framework *via* several inter­molecular O—H⋯O hydrogen bonds.

## Related literature

For related literature, see: Aghabozorg *et al.* (2007 ▶); Baruah *et al.* (2007 ▶); Drew *et al.* (1971 ▶); Ghoer & Youssef (1993 ▶); Kang *et al.* (2006 ▶); Li *et al.* (2006 ▶); Manteghi *et al.* (2007 ▶); Patrick *et al.* (2003 ▶); Sun *et al.* (2006 ▶); Takusagawa & Koetzle (1978 ▶); Turner & Batten (2007 ▶); Zhang & You (2003 ▶); Zhang *et al.* (2003 ▶).
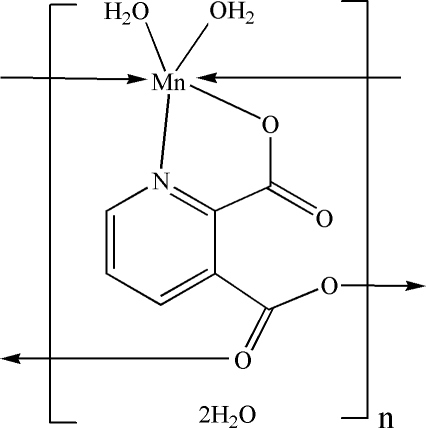


## Experimental

### 

#### Crystal data

[Mn(C_7_H_3_NO_4_)(H_2_O)_2_]·2H_2_O
*M* *_r_* = 292.11
Monoclinic, 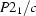

*a* = 6.5719 (8) Å
*b* = 7.6703 (9) Å
*c* = 20.566 (3) Å
β = 93.3540 (10)°
*V* = 1034.9 (2) Å^3^
*Z* = 4
Mo *K*α radiation
μ = 1.31 mm^−1^
*T* = 291 (2) K
0.38 × 0.15 × 0.11 mm

#### Data collection

Bruker APEXII CCD area-detector diffractometer
Absorption correction: multi-scan (*SADABS*; Sheldrick, 1996 ▶) *T*_min_ = 0.639, *T*_max_ = 0.865
6428 measured reflections
1921 independent reflections
1774 reflections with *I* > 2σ(*I*)
*R*_int_ = 0.015

#### Refinement

*R*[*F* ^2^ > 2σ(*F* ^2^)] = 0.035
*wR*(*F* ^2^) = 0.095
*S* = 1.07
1921 reflections
154 parameters
3 restraints
H-atom parameters constrained
Δρ_max_ = 0.49 e Å^−3^
Δρ_min_ = −0.74 e Å^−3^

### 

Data collection: *APEX2* (Bruker, 2004 ▶); cell refinement: *APEX2*; data reduction: *SAINT* (Bruker, 2004 ▶); program(s) used to solve structure: *SHELXS97* (Sheldrick, 2008 ▶); program(s) used to refine structure: *SHELXL97* (Sheldrick, 2008 ▶); molecular graphics: *SHELXTL* (Sheldrick, 2008 ▶); software used to prepare material for publication: *SHELXTL*.

## Supplementary Material

Crystal structure: contains datablocks global, I. DOI: 10.1107/S1600536808029413/sg2261sup1.cif

Structure factors: contains datablocks I. DOI: 10.1107/S1600536808029413/sg2261Isup2.hkl

Additional supplementary materials:  crystallographic information; 3D view; checkCIF report

## Figures and Tables

**Table:** 

Mn1—O3	2.098 (3)
Mn1—O4	2.139 (3)
Mn1—O8^i^	2.144 (2)
Mn1—O5	2.160 (3)
Mn1—O6^ii^	2.242 (3)
Mn1—N1^i^	2.263 (3)

**Table:** 

O3—Mn1—O4	96.07 (11)
O3—Mn1—O8^i^	168.80 (10)
O3—Mn1—O5	87.98 (10)
O4—Mn1—O5	87.54 (10)
O8^i^—Mn1—O5	95.89 (9)
O3—Mn1—O6^ii^	84.63 (10)
O5—Mn1—O6^ii^	164.11 (10)
O4—Mn1—N1^i^	166.71 (11)

Symmetry codes: (i) 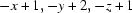; (ii) 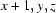.

**Table 2:** Hydrogen-bond geometry (Å, °)

*D*—H⋯*A*	*D*—H	H⋯*A*	*D*⋯*A*	*D*—H⋯*A*
O1—H1*W*⋯O8	0.84	1.95	2.793 (4)	177
O1—H2*W*⋯O2^iii^	0.84	2.09	2.845 (4)	149
O2—H4*W*⋯O6^iv^	0.84	2.07	2.884 (4)	165
O2—H4*W*⋯O4^v^	0.84	2.56	3.043 (4)	118
O2—H3*W*⋯O4^vi^	0.85	1.94	2.788 (4)	180
O3—H5*W*⋯O7^iv^	0.84	1.85	2.691 (4)	175
O3—H6*W*⋯O1^iv^	0.85	1.92	2.730 (4)	159
O4—H8*W*⋯O1^i^	0.85	2.61	3.457 (4)	180
O4—H7*W*⋯O1^vii^	0.84	1.86	2.691 (4)	168
O4—H8*W*⋯O2^vii^	0.85	2.37	2.788 (4)	111

Symmetry codes: (i) 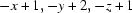; (iii) 
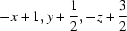
; (iv) 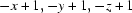; (v) 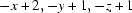; (vi) 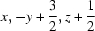; (vii) 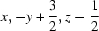.

**Fig. 1.**
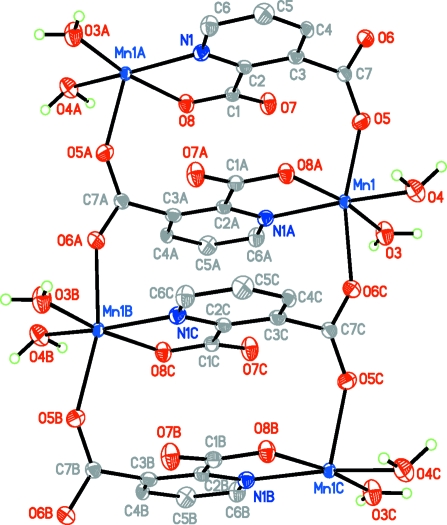
The structure of the building unit of the one-dimensional of (I), with the atom-numbering scheme. Displacement ellipsoids are drawn at the 30% probability level. Uncoordinate water molecules and H atoms on C atoms have been omitted. [Symmetry codes: (A) 2 - x, 2 - y, 1 - z; (B) 1 - x, 2 - y, 1 - z; (C) -1 + x, y, z.]

**Fig. 2.**
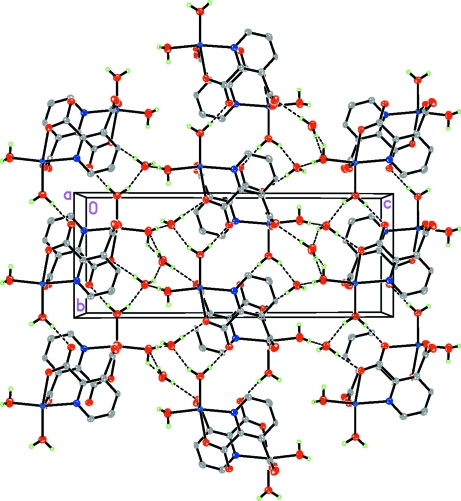
The crystal packing of (I), showing hydrogen bonds as dashed lines. For the sake of clarity, H atoms on C atoms have been omitted.

## supplementary crystallographic information

### Comment

Pyridine-2,3-dicarboxylic acid (H_2_PDC), being a potential multidentate bridging ligand, has aroused considerable interests in recent decades and a number of metal complexes have been reported. In these complexes, pyridine-2,3-dicarboxylic acid is partly or fully deprotonated and shows diverse coordination modes, such as non-coordinate (Takusagawa & Koetzle, 1978; Manteghi *et al.*, 2007), monodentate (Drew *et al.*, 1971; Ghoer & Youssef, 1993; Patrick *et al.*, 2003; Baruah *et al.*, 2007), µ_2_-bridging (Zhang *et al.*, 2003; Aghabozorg *et al.*, 2007; Sun *et al.*, 2006; Turner & Batten, 2007; Kang *et al.*, 2006), µ_3_-bridging (Zhang & You, 2003; Li *et al.*, 2006). Here we describe another new compound in which the PDC is µ_3_-bridging, (I),(Fig. 1).

Complex (I) is composed of {[Mn(C_7_H_3_NO_4_)(H_2_O)_2_].2H_2_O}_n_ units, in which the Mn^II^ ion is six-coordinated in a distorted octahedral geometry (Table 1) formed by two coordinated water molecules, one N and one O atoms of a PDC dianion and two different carboxylate O atoms in the axial position from another two adjacent PDC ligands. The deprotonated PDC are µ_3_-bridging ligands and they join the neighbouring Mn^II^ ions to form this one-dimensional linear chain structure along *a* axis. This kind of µ_3_-bridging mode is different from that have been published (Zhang & You, 2003; Li *et al.*, 2006) as in this paper one bridging carboxylate O atom only links one metal ions, while in the latter one bridging carboxylate O atom simultaneously links two metal ions,respectively.

The carboxylate O atoms of PDC dianion and coordinate and non coordinated water molecules are all involved in rich O—H···O intermolecular hydrogen bonds (Table 2) and they connect polymetric chains into a three-dimensional framework (Fig. 2).

### Experimental

The ligand H_2_PDC (1 mmol, 0.17 g) and NaOH (2 mmol, 0.08 g) were dissolved in water and methanol mixed solvent (20 ml, *v*/*v* 1:1). To this solution, Mn(CH_3_COO)_2_.4H_2_O (1 mmol, 0.25 g) was added and the resulting mixture was stirred and refluxed at 343 K for 5 h, then cooled to room temperature. After filtration and evaporation in air for a week, pink block-shaped crystals were obtained in a yield of 37%. Analysis, found (%): C, 28.70; H, 3.80; N, 4.71. C_7_H_11_Mn N O_8_ requires (%): C,28.75; H,3.76; N,4.79. (CCDC number 668395)

### Refinement

H atoms bonded to C atoms were positioned geometrically with C—H distance of 0.93 Å, and treated as riding atoms, with *U*_iso_(H) = 1.2*U*_eq_. H atoms bonded to O atoms were located in a difference Fourier map and refined isotropically.

### Crystal data

**Table:**

[Mn(C_7_H_3_NO_4_)(H_2_O)_2_]·2H_2_O	*F*(000) = 596
*M**_r_* = 292.11	*D*_x_ = 1.875 Mg m^−^^3^
Monoclinic, *P*2_1_/*c*	Mo *K*α radiation, λ = 0.71073 Å
Hall symbol: -P 2ybc	Cell parameters from 4039 reflections
*a* = 6.5719 (8) Å	θ = 2.8–28.1°
*b* = 7.6703 (9) Å	µ = 1.31 mm^−^^1^
*c* = 20.566 (3) Å	*T* = 291 K
β = 93.354 (1)°	Block, pink
*V* = 1034.9 (2) Å^3^	0.38 × 0.15 × 0.11 mm
*Z* = 4	

### Data collection

**Table:**

Bruker APEXII CCD area-detector diffractometer	1921 independent reflections
Radiation source: fine-focus sealed tube	1774 reflections with *I* > 2σ(*I*)
graphite	*R*_int_ = 0.015
φ and ω scans	θ_max_ = 25.5°, θ_min_ = 2.8°
Absorption correction: multi-scan (SADABS; Sheldrick, 1996)	*h* = −7→7
*T*_min_ = 0.640, *T*_max_ = 0.865	*k* = −9→9
6428 measured reflections	*l* = −23→24

### Refinement

**Table:**

Refinement on *F*^2^	Primary atom site location: structure-invariant direct methods
Least-squares matrix: full	Secondary atom site location: difference Fourier map
*R*[*F*^2^ > 2σ(*F*^2^)] = 0.035	Hydrogen site location: inferred from neighbouring sites
*wR*(*F*^2^) = 0.095	H-atom parameters constrained
*S* = 1.07	*w* = 1/[σ^2^(*F*_o_^2^) + (0.042*P*)^2^ + 2.0761*P*] where *P* = (*F*_o_^2^ + 2*F*_c_^2^)/3
1921 reflections	(Δ/σ)_max_ = 0.001
154 parameters	Δρ_max_ = 0.49 e Å^−^^3^
3 restraints	Δρ_min_ = −0.74 e Å^−^^3^

### Special details

**Table:**

Geometry. All e.s.d.'s (except the e.s.d. in the dihedral angle between two l.s. planes) are estimated using the full covariance matrix. The cell e.s.d.'s are taken into account individually in the estimation of e.s.d.'s in distances, angles and torsion angles; correlations between e.s.d.'s in cell parameters are only used when they are defined by crystal symmetry. An approximate (isotropic) treatment of cell e.s.d.'s is used for estimating e.s.d.'s involving l.s. planes.
Refinement. Refinement of *F*^2^ against ALL reflections. The weighted *R*-factor *wR* and goodness of fit *S* are based on *F*^2^, conventional *R*-factors *R* are based on *F*, with *F* set to zero for negative *F*^2^. The threshold expression of *F*^2^ > σ(*F*^2^) is used only for calculating *R*-factors(gt) *etc*. and is not relevant to the choice of reflections for refinement. *R*-factors based on *F*^2^ are statistically about twice as large as those based on *F*, and *R*-factors based on ALL data will be even larger.

### Fractional atomic coordinates and isotropic or equivalent isotropic displacement parameters (Å^2^)

**Table:**

	*x*	*y*	*z*	*U*_iso_*/*U*_eq_	
Mn1	0.69176 (6)	0.76827 (6)	0.38821 (2)	0.01743 (17)	
O1	0.3592 (5)	0.7546 (3)	0.70493 (14)	0.0449 (7)	
H1W	0.3455	0.8191	0.6719	0.067*	
H2W	0.2695	0.7814	0.7307	0.067*	
O2	0.9617 (5)	0.4500 (4)	0.74324 (14)	0.0543 (8)	
H3W	0.8800	0.5269	0.7561	0.081*	
H4W	0.9878	0.3937	0.7099	0.081*	
O3	0.7081 (4)	0.4951 (3)	0.38849 (13)	0.0437 (7)	
H5W	0.7244	0.4234	0.4193	0.066*	
H6W	0.6950	0.4383	0.3532	0.066*	
O4	0.6927 (4)	0.7979 (4)	0.28481 (13)	0.0420 (6)	
H7W	0.5997	0.7749	0.2562	0.063*	
H8W	0.6803	0.9080	0.2873	0.063*	
O5	0.3643 (4)	0.7440 (3)	0.37627 (14)	0.0374 (6)	
O6	0.0271 (4)	0.7556 (3)	0.37392 (13)	0.0362 (6)	
O7	0.2349 (5)	0.7489 (3)	0.51820 (13)	0.0393 (6)	
O8	0.3057 (4)	0.9571 (3)	0.59238 (11)	0.0313 (5)	
N1	0.2620 (4)	1.2081 (4)	0.50222 (14)	0.0272 (6)	
C1	0.2629 (5)	0.9023 (4)	0.53482 (15)	0.0251 (7)	
C2	0.2440 (4)	1.0408 (4)	0.48204 (15)	0.0234 (6)	
C3	0.2117 (5)	1.0010 (4)	0.41594 (16)	0.0257 (7)	
C4	0.1928 (4)	1.1277 (4)	0.37076 (14)	0.0212 (6)	
H4	0.1690	1.1020	0.3268	0.025*	
C5	0.2096 (6)	1.2911 (5)	0.39172 (18)	0.0374 (9)	
H5	0.1971	1.3810	0.3614	0.045*	
C6	0.2452 (6)	1.3332 (5)	0.45735 (17)	0.0331 (8)	
H6	0.2574	1.4495	0.4699	0.040*	
C7	0.2003 (5)	0.8171 (5)	0.38797 (15)	0.0255 (7)	

### Atomic displacement parameters (Å^2^)

**Table:**

	*U*^11^	*U*^22^	*U*^33^	*U*^12^	*U*^13^	*U*^23^
Mn1	0.0193 (3)	0.0159 (3)	0.0170 (3)	−0.00007 (16)	0.00015 (17)	−0.00174 (16)
O1	0.0536 (18)	0.0450 (16)	0.0354 (15)	0.0076 (13)	−0.0033 (13)	0.0040 (12)
O2	0.0584 (19)	0.0623 (19)	0.0426 (16)	0.0277 (16)	0.0062 (13)	−0.0098 (14)
O3	0.0653 (19)	0.0273 (13)	0.0376 (15)	−0.0005 (12)	−0.0048 (13)	0.0011 (11)
O4	0.0416 (15)	0.0498 (16)	0.0339 (14)	−0.0068 (13)	−0.0048 (11)	0.0014 (12)
O5	0.0277 (13)	0.0371 (14)	0.0475 (16)	0.0024 (10)	0.0035 (11)	−0.0113 (11)
O6	0.0259 (13)	0.0410 (15)	0.0413 (15)	−0.0027 (10)	−0.0015 (11)	−0.0103 (11)
O7	0.0592 (18)	0.0225 (13)	0.0353 (14)	−0.0028 (11)	−0.0030 (13)	0.0002 (10)
O8	0.0405 (14)	0.0286 (12)	0.0242 (12)	0.0002 (10)	−0.0024 (10)	0.0017 (10)
N1	0.0272 (14)	0.0247 (13)	0.0297 (15)	0.0008 (11)	0.0024 (11)	−0.0001 (11)
C1	0.0223 (16)	0.0250 (16)	0.0279 (16)	0.0009 (12)	0.0010 (12)	−0.0004 (13)
C2	0.0182 (15)	0.0249 (16)	0.0273 (16)	0.0004 (12)	0.0016 (12)	0.0000 (13)
C3	0.0183 (15)	0.0292 (17)	0.0298 (17)	−0.0003 (12)	0.0016 (12)	−0.0001 (13)
C4	0.0262 (16)	0.0241 (15)	0.0133 (13)	0.0011 (12)	−0.0004 (11)	0.0015 (11)
C5	0.046 (2)	0.0328 (19)	0.033 (2)	0.0036 (16)	0.0031 (16)	0.0099 (15)
C6	0.043 (2)	0.0233 (16)	0.0325 (18)	−0.0003 (14)	0.0019 (15)	0.0017 (14)
C7	0.0232 (17)	0.0311 (17)	0.0220 (15)	0.0000 (13)	0.0002 (12)	−0.0007 (13)

### Geometric parameters (Å, °)

**Table:**

Mn1—O3	2.098 (3)	O6—Mn1^iii^	2.243 (2)
Mn1—O4	2.139 (3)	O7—C1	1.236 (4)
Mn1—O8^i^	2.144 (2)	O8—C1	1.272 (4)
Mn1—O5	2.160 (3)	O8—Mn1^i^	2.144 (2)
Mn1—O6^ii^	2.242 (3)	N1—C6	1.332 (4)
Mn1—N1^i^	2.263 (3)	N1—C2	1.352 (4)
O1—H1W	0.8416	N1—Mn1^i^	2.263 (3)
O1—H2W	0.8409	C1—C2	1.519 (4)
O2—H3W	0.8502	C2—C3	1.397 (4)
O2—H4W	0.8369	C3—C4	1.346 (4)
O3—H5W	0.8410	C3—C7	1.523 (5)
O3—H6W	0.8474	C4—C5	1.327 (5)
O4—H7W	0.8414	C4—H4	0.9300
O4—H8W	0.8497	C5—C6	1.394 (5)
O5—C7	1.250 (4)	C5—H5	0.9300
O6—C7	1.250 (4)	C6—H6	0.9300
			
O3—Mn1—O4	96.07 (11)	C1—O8—Mn1^i^	119.8 (2)
O3—Mn1—O8^i^	168.80 (10)	C6—N1—C2	118.0 (3)
O4—Mn1—O8^i^	94.60 (10)	C6—N1—Mn1^i^	129.3 (2)
O3—Mn1—O5	87.98 (10)	C2—N1—Mn1^i^	112.7 (2)
O4—Mn1—O5	87.54 (10)	O7—C1—O8	126.4 (3)
O8^i^—Mn1—O5	95.89 (9)	O7—C1—C2	117.6 (3)
O3—Mn1—O6^ii^	84.63 (10)	O8—C1—C2	116.0 (3)
O4—Mn1—O6^ii^	79.30 (10)	N1—C2—C3	120.8 (3)
O8^i^—Mn1—O6^ii^	94.02 (9)	N1—C2—C1	116.3 (3)
O5—Mn1—O6^ii^	164.11 (10)	C3—C2—C1	122.9 (3)
O3—Mn1—N1^i^	94.21 (10)	C4—C3—C2	121.1 (3)
O4—Mn1—N1^i^	166.71 (11)	C4—C3—C7	114.1 (3)
O8^i^—Mn1—N1^i^	74.75 (9)	C2—C3—C7	124.8 (3)
O5—Mn1—N1^i^	101.24 (10)	C5—C4—C3	117.1 (3)
O6^ii^—Mn1—N1^i^	93.33 (10)	C5—C4—H4	121.5
H1W—O1—H2W	108.6	C3—C4—H4	121.5
H3W—O2—H4W	140.9	C4—C5—C6	122.6 (3)
Mn1—O3—H5W	131.3	C4—C5—H5	118.7
Mn1—O3—H6W	120.6	C6—C5—H5	118.7
H5W—O3—H6W	108.1	N1—C6—C5	120.4 (3)
Mn1—O4—H7W	128.9	N1—C6—H6	119.8
Mn1—O4—H8W	92.3	C5—C6—H6	119.8
H7W—O4—H8W	100.4	O6—C7—O5	124.7 (3)
C7—O5—Mn1	143.5 (2)	O6—C7—C3	117.4 (3)
C7—O6—Mn1^iii^	147.3 (2)	O5—C7—C3	117.6 (3)
			
O3—Mn1—O5—C7	151.0 (4)	N1—C2—C3—C7	176.7 (3)
O4—Mn1—O5—C7	−112.9 (4)	C1—C2—C3—C7	−2.7 (5)
O8^i^—Mn1—O5—C7	−18.5 (4)	C2—C3—C4—C5	1.1 (5)
O6^ii^—Mn1—O5—C7	−146.8 (4)	C7—C3—C4—C5	−177.4 (3)
N1^i^—Mn1—O5—C7	57.1 (4)	C3—C4—C5—C6	0.0 (5)
Mn1^i^—O8—C1—O7	−171.8 (3)	C2—N1—C6—C5	0.2 (5)
Mn1^i^—O8—C1—C2	7.9 (4)	Mn1^i^—N1—C6—C5	−177.7 (3)
C6—N1—C2—C3	0.9 (5)	C4—C5—C6—N1	−0.7 (6)
Mn1^i^—N1—C2—C3	179.1 (2)	Mn1^iii^—O6—C7—O5	165.6 (3)
C6—N1—C2—C1	−179.7 (3)	Mn1^iii^—O6—C7—C3	−20.0 (6)
Mn1^i^—N1—C2—C1	−1.4 (3)	Mn1—O5—C7—O6	−177.6 (3)
O7—C1—C2—N1	175.7 (3)	Mn1—O5—C7—C3	8.0 (6)
O8—C1—C2—N1	−4.0 (4)	C4—C3—C7—O6	−81.4 (4)
O7—C1—C2—C3	−4.8 (5)	C2—C3—C7—O6	100.2 (4)
O8—C1—C2—C3	175.5 (3)	C4—C3—C7—O5	93.4 (4)
N1—C2—C3—C4	−1.6 (5)	C2—C3—C7—O5	−85.0 (4)
C1—C2—C3—C4	179.0 (3)		

Symmetry codes: (i) −*x*+1, −*y*+2, −*z*+1; (ii) *x*+1, *y*, *z*; (iii) *x*−1, *y*, *z*.

### Hydrogen-bond geometry (Å, °)

**Table:**

*D*—H···*A*	*D*—H	H···*A*	*D*···*A*	*D*—H···*A*
O1—H1W···O8	0.84	1.95	2.793 (4)	177.
O1—H2W···O2^iv^	0.84	2.09	2.845 (4)	149.
O2—H4W···O6^v^	0.84	2.07	2.884 (4)	165.
O2—H4W···O4^vi^	0.84	2.56	3.043 (4)	118.
O2—H3W···O4^vii^	0.85	1.94	2.788 (4)	180.
O3—H5W···O7^v^	0.84	1.85	2.691 (4)	175.
O3—H6W···O1^v^	0.85	1.92	2.730 (4)	159.
O4—H8W···O1^i^	0.85	2.61	3.457 (4)	180.
O4—H7W···O1^viii^	0.84	1.86	2.691 (4)	168.
O4—H8W···O2^viii^	0.85	2.37	2.788 (4)	111.

Symmetry codes: (iv) −*x*+1, *y*+1/2, −*z*+3/2; (v) −*x*+1, −*y*+1, −*z*+1; (vi) −*x*+2, −*y*+1, −*z*+1; (vii) *x*, −*y*+3/2, *z*+1/2; (i) −*x*+1, −*y*+2, −*z*+1; (viii) *x*, −*y*+3/2, *z*−1/2.
